# Fibrin-targeted phase shift microbubbles for the treatment of microvascular obstruction

**DOI:** 10.7150/ntno.85092

**Published:** 2024-01-01

**Authors:** Soheb Anwar Mohammed, Muhammad Wahab Amjad, Maria F. Acosta, Xucai Chen, Linda Lavery, Dillon Hanrahan, Evan C. Unger, Emmanuelle J. Meuillet, John J. Pacella

**Affiliations:** 1 Center for Ultrasound Molecular Imaging and Therapeutics, Heart and Vascular Medicine Institute, University of Pittsburgh. 200 Lothrop St, Pittsburgh, PA, USA.; 2 Microvascular Therapeutics (MVT), Inc. 1635 E. 18 th Street, Tucson, AZ, USA.

**Keywords:** fibrin-targeted microbubbles, microvascular obstruction, cavitation, phase-shift microbubbles, sonoreperfusion

## Abstract

**Rationale:** Microvascular obstruction (MVO) following percutaneous coronary intervention (PCI) is a common problem associated with adverse clinical outcomes. We are developing a novel treatment, termed sonoreperfusion (SRP), to restore microvascular patency. This entails using ultrasound-targeted microbubble cavitation (UTMC) of intravenously administered gas-filled lipid microbubbles (MBs) to dissolve obstructive microthrombi in the microvasculature. In our prior work, we used standard-sized lipid MBs. In the present study, to improve upon the efficiency and efficacy of SRP, we sought to determine the therapeutic efficacy of fibrin-targeted phase shift microbubbles (FTPSMBs) in achieving successful reperfusion of MVO. We hypothesized that owing to their much smaller size and affinity for thrombus, FTPSMBs would provide more effective dissolution of microthrombi when compared to that of the corresponding standard-sized lipid MBs.

**Methods:** MVO in the rat hindlimb was created by direct injection of microthrombi into the left femoral artery. Definity MBs (Lantheus Medical Imaging) were infused through the jugular vein for contrast-enhanced ultrasound imaging (CEUS). A transducer was positioned vertically above the hindlimb for therapeutic US delivery during the concomitant administration of various therapeutic formulations, including (1) un-targeted MBs; (2) un-targeted phase shift microbubbles (PSMBs); (3) fibrin-targeted MB (FTMBs); and (4) fibrin-targeted PSMBs (FTPSMBs). CEUS cine loops with burst replenishment were obtained at baseline (BL), 10 min post-MVO, and after each of two successive 10-minute SRP treatment sessions (TX1, TX2) and analyzed (MATLAB).

**Results:** *In-vitro* binding affinity assay showed increased fibrin binding peptide (FBP) affinity for the fibrin clots compared with the untargeted peptide (DK12). Similarly, in our *in-vitro* model of MVO, we observed a higher binding affinity of fluorescently labeled FTPSMBs with the porcine microthrombi compared to FTMBs, PSMBs, and MBs. Finally, in our hindlimb model, we found that UTMC with FTPSMBs yielded the greatest recovery of blood volume (dB) and flow rate (dB/sec) following MVO, compared to all other treatment groups.

**Conclusions:** SRP with FTPSMBs achieves more rapid and complete reperfusion of MVO compared to FTMBs, PSMBs, and MBs. Studies to explore the underlying physical and molecular mechanisms are underway.

## Introduction

Cardiovascular disease is the leading cause of morbidity and mortality in the United States 1. More than 1 million Americans are estimated to have a new or recurrent acute myocardial infarction (AMI) annually 2. The contemporary treatment for AMI is percutaneous coronary intervention (PCI), which aims to restore perfusion to the myocardium through recanalization of the epicardial vessels to effect maximal myocardial salvage 3. Although the mortality from AMI has decreased in recent years, post-MI congestive heart failure is increasing due to a phenomenon termed microvascular obstruction (MVO), which ultimately limits myocardial salvage 4.

MVO results in myocardial microvascular hypoperfusion post-PCI, despite patent upstream epicardial vessels. This occurs due to distal micro-embolization, ischemia-reperfusion injury, activation of inflammatory cascades, and tissue edema, ultimately resulting in vessel compression and local vasospasm 5-11. It has been estimated that up to 60% of all acute ST-segment elevation myocardial infarction (STEMI) patients receiving PCI develop MVO. This results in decreased left ventricular (LV) function and major adverse cardiac events, including cardiac death, stroke, myocardial infarction, and heart failure 12-14. MVO is linked with increased infarct size and adverse LV remodelling, with MVO persistence a stronger predictor for functional recovery than the transmural infarct extension 14-18. Conventional strategies for treating or preventing MVO include the administration of vasodilators and antiplatelet therapy, thrombus aspiration, embolic protection devices, and even the use of hyperoxemic intracoronary reperfusion therapy 19. Even with these strategies, no definitive therapeutic consensus for MVO exists, and many clinical trials have yielded conflicting results 5,7,13.

Therefore, we are addressing an urgent unmet need by devising an image-guided acute therapy, termed 'sonoreperfusion' (SRP), that resolves MVO and limits its adverse sequelae. This technique involves ultrasound-targeted microbubble cavitation (UTMC) of intravenously injected lipid MBs, resulting in MB oscillation (cavitation), generating intravascular shear forces which cause dissolution of the microthrombi, resulting in reperfusion of MVO 20,21. UTMC instigates both mechanical effects and signaling actions, disrupting microthrombi and activating endothelial nitric oxide (NO) pathways for local vasodilation to restore perfusion 20,22,23. Therapeutic ultrasound (US) for reperfusion is a rapidly evolving field of investigation. We have demonstrated the efficacy of UTMC for the treatment of MVO and improving the tissue perfusion 21,22. In addition to thrombolysis, UTMC may also be used as a means for the targeted delivery of therapeutics by loaded MBs. This promotes site-specific concentration of therapeutic agents resulting in higher efficiency than systemic administration and reducing off-target effects 24-29. Thus, SRP is an ideal solution for treating MVO because it is a minimally invasive theranostic technique that visualizes regions of MVO using MBs contrast agents and then provides image-guided therapeutic US pulses.

In contrast to smaller caliber PSMBs, standard-sized lipid MBs have more limited penetration into thrombus structure, imposed by the average fibrin spacing and MB size 30,31. This steric limitation would inhibit agent-thrombus penetration and would prevent maximal mechanical lysis. Likewise, non-targeted agents have less thrombus affinity compared to targeted agents. This reduced proximity of the therapeutic agent to the thrombus would also result in less effective sonothrombolysis. To improve the efficiency and efficacy of our SRP technique, we have developed novel fibrin-targeted phase shift microbubbles (FTPSMBs) by incorporating a peptide ligand targeting fibrin to (1) provide greater thrombus penetration due to less steric hindrance with the smaller caliber PSMBs and (2) enhance thrombus affinity via fibrin targeting. Taken together, we hypothesized that this approach would allow enhanced access of FTMBs/FTPSMBs to the microthrombi, resulting in improved transmission of shear stress induced by FTMBs/FTPSMBs oscillations, more mechanical lysis of microthrombi and thus enhanced reperfusion.

## Materials and methods

### Materials

All phospholipids, dipalmitoylphosphatidylcholine (DPPC), dipalmitoylphosphatidylethanolamine (DPPE), 1,2-dipalmitoyl-sn-glycero-3-phosphoethanolamine-N-[methoxy(polyethylene glycol)-5000] (ammonium salt) (DPPE-MPEG-5000), and 1,2-distearoyl-sn-glycero-3-phosphoethanolamine-N- methoxy(polyethylene glycol)-5000 (DSPE-MPEG-5000) were purchased from Avanti Polar Lipids (Birmingham, Al). Sodium hydroxide (NaOH), sodium phosphate monobasic monohydrate (NaH_2_PO_4_), sodium phosphate dibasic heptahydrate, glycerol, and propylene glycol, were obtained from Sigma-Aldrich (St. Louis, MO). Fibrin binding peptide (FBP) was acquired from LifeproTein®. Heparin, Thioflavin T, Tris base, EDTA, DTT, fibrin, fibrinogen, thrombin, and 5(6)-carboxy-tetramethylrhodamine N-succinimidyl ester (Rh), were from Sigma-Aldrich, USA. Octafluoropropane gas (OFP, C_3_F_8_) was purchased from FluoroMed, L.P, USA.

### Formation of the Fibrin Peptide Bioconjugates

**Figure 2:**
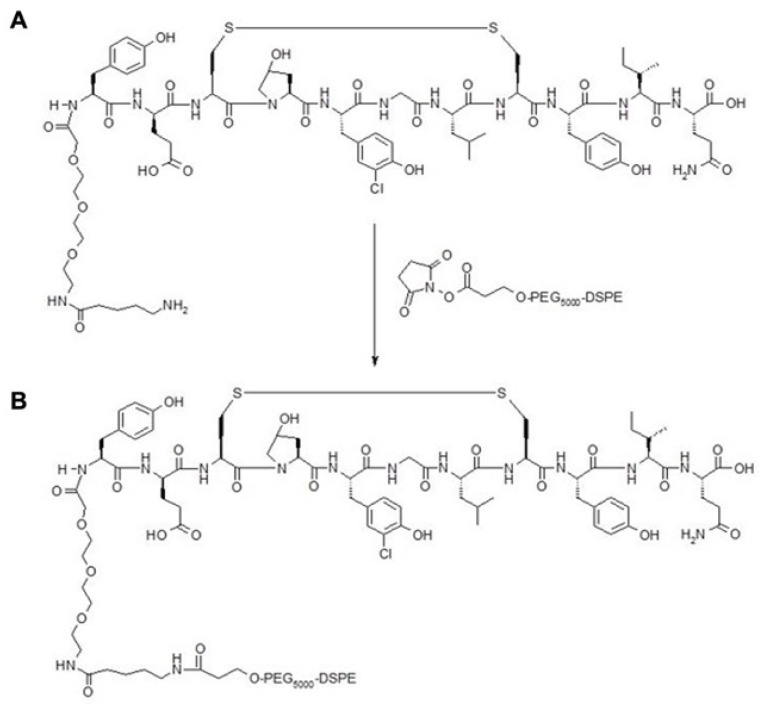
Fibrin Binding Peptide with an amine functional group (A) conjugated to DSPE-PEG5000-NHS Ester to make a product with an amide linker (B).

The fibrin binding peptide (FBP) was initially developed by Caravan et al. as a magnetic resonance contrast agent 32,33. The core peptide was obtained from a commercial supplier of peptides (LifeproTein® LLC, USA). DK-12 is a control peptide (12 amino acids long with an isoelectric point of 3.36). The peptides with a mini-PEG linker and amine functional group was conjugated to [(succinimidyloxyglutaryl)aminopropyl, polyethyleneglycol-5000]-carbamyl distearoylphosphatidyl-ethanolamine (sodium salt) (DSPE-PEG5000-NHS ester) to synthesize a product with an amide linker (Figure 2). The product was purified with High-Pressure Liquid Chromatography (HPLC) and characterized with a Mass Spectroscopy (MS) instrument. The final bioconjugate was named DSPE-PEG5000-FBP or DSPE-PEG5000-DK12.

### Preparation of the Control MBs Formulation

Each mL of the clear liquid contains 0.75 mg of lipid blend consisting of 0.045 DPPE, 0.401mg DPPC and 0.304 mg DPPE-MPEG-5000; 103.5 mg propylene glycol, 126.2 mg glycerin, 2.34 mg sodium phosphate monobasic monohydrate, 2.16 mg sodium phosphate dibasic heptahydrate, and 4.87 mg sodium chloride in Water for Injection. The pH is 6.2-6.8.

Briefly, the lipids, suspended in propylene glycol, were heated to 70±5 °C until they dissolved. The lipid solution was then added to an aqueous solution containing sodium chloride, phosphate buffer, and glycerol and allowed to mix thoroughly by stirring (at 70±5 °C). The solution was introduced into 2.0 mL Wheaton (VWR, Radnor, USA) lyophilization vials (1.5 mL fill volume). OFP gas was injected into each vial. The vials were then crimped and sealed.

### Production of the Non-targeted and the Fibrin-targeted Microbubbles and PSMBs

**Figure 3:**
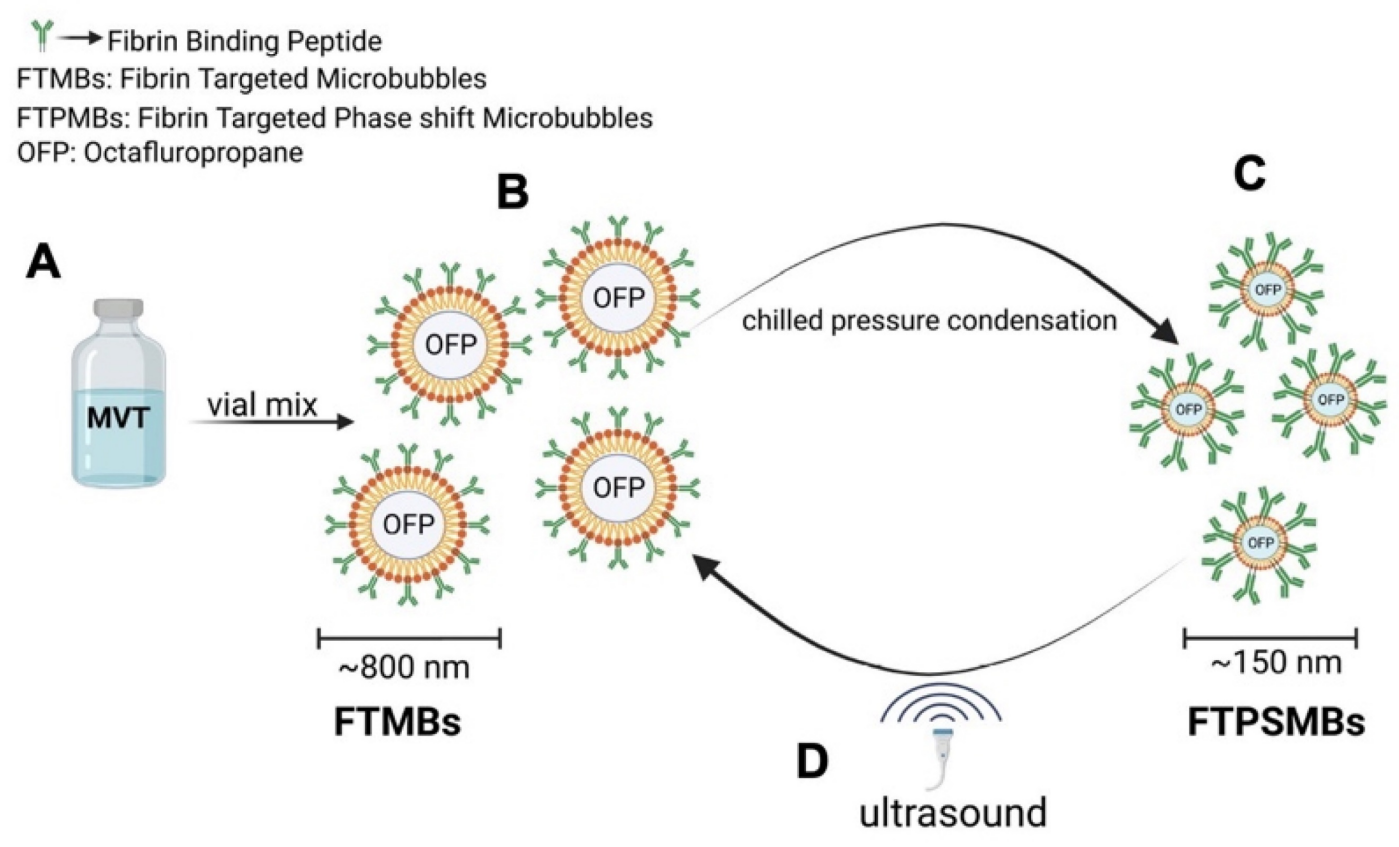
** Illustration of phase shift in the presence of ultrasound: (A)** Ingredients of fibrin-targeted microbubbles (FTMBs) in solution inactive state. (B) Upon vial mix for 45 sec results in FTMBs having a gaseous core shell of octafluoropropane. (C) Chilled pressure condensation of FTMBs results in the production of FTPSMBs. (D) Under the influence of ultrasound FTPSMBs phase shift to FTMBs. (Figure created by BioRender)

To produce the Fibrin-targeted MBs and PSMBs (FTMBs and FTPSMBs), 1%, 0.1%, or 0.01% of the bioconjugate were mixed with 81%, 81.9%, or 81.99% of DPPC (respectively), 8% of DPPE-MPEG (5000) and 10% of DPPE. For the non-targeted (control) MBs and PSMBs, the bioconjugate DSPE-PEG5000-FBP was replaced with N-(Carbonyl-methoxy polyethylene glycol 5000)- carbamyl distearoylphosphatidyl-ethanolamine (sodium salt) (DSPE-MPEG5000), everything else remained the same. To form MBs/FTMBs of all formulations, the vials were activated with the VialMix™ for 45 s. In the FTMBs/MBs activation process, the OFP gas is entrapped in the core-shell of the FTMBs/MBs, resulting in 6.78x10^8^/mL of FTMBs and 3.43x10^9^/mL of MBs. After this, the activated FTMBs/MBs were cooled down at -15 °C to -20 °C for 5 min. The chilled FTMBs/MBs were withdrawn with a 3 ml syringe and locked with a stopcock. The syringe was chilled for 5 minutes at -15°C to -20°C. Then, the FTMBs/MBs were condensed into FTPSMBs/PSMBs by applying pressure for 5 min. During this procedure, OFP is converted from a gaseous state to a liquid state in the lipid core-shell. However, in the presence of ultrasound, FTPSMBs/PSMBs are phase-shifted to FTMBs/MBs (Figure 3).

### Production of the Non-targeted and the Targeted MBs and PSMBs with DiO Dye

To produce the Fibrin-targeted MBs and PSMBs (FTMBs and FTPSMBs), 1%, 0.1%, or 0.01% mol of the bioconjugate were mixed with 81%, 81.9%, or 81.99% mol of DPPC (respectively), 8% mol of DPPE-MPEG5000 and 10% mole of DPPE. For the non-targeted (control) MBs and PSMBs, the bioconjugate DSPE-PEG5000-FBP was replaced with N-(Carbonyl-methoxypolyethyleneglycol 5000)- carbamyl distearoylphosphatidyl-ethanolamine (sodium salt) (DSPE-MPEG5000). The lipids, suspended in propylene glycol, were gradually heated until they dissolved. 1% w/v of DiO dye was added to the lipid mixture. The above-described method was followed to activate the DiO dye-loaded MBs and PSMBs.

### *In-vitro* Fibrin Binding Assay

FBP was tagged with 5(6)-carboxytetramethylrhodamine N-succinimidyl ester (Rh, MW= 527.52 Da) fluorescent dye. DK-12, a peptide with 12 amino acids and MW= 1770.05 Da, was also tagged with Rh fluorescent dye as the control. Products of the conjugation step were purified with high-performance liquid chromatography (HPLC) and characterized with mass spectrometry (MS). After it was confirmed that both peptides were successfully tagged with the fluorescent dye, these fluorescently labeled peptides were tested for their ability to bind fibrin. Briefly, the bottom of a dark 96-well plate was coated with fibrin (obtained from the fibrinogen+thrombin reaction after 72 h at room temperature). Increasing concentrations of the peptide was added and incubated for 1 h. Wells were washed three times with PBS. Finally, the fluorescence was measured at λex 485 nm; and λem 528 nm.

### *In-vitro* Physical and Chemical Characterization

**Particle sizing:** Post-activation sizing of MBs was performed on the Nicomp 780 sampling in 128 channels (Particle Sizing Systems, Ft. Richey, FL). MBs vials were activated and diluted in filtered normal saline using a 100 µL pipet. PSMBs preparations were studied for particle sizing using a Malvern Zetasizer Nano ZS from Malvern Panalytical Inc. USA. Ten droplets of the PSMBs solution were diluted in filtered normal saline.

**Gas content:** The OFP content pre-activation was measured by Gas Chromatography using an Agilent 7890B Gas Chromatograph equipped with a PAL 3 Autosampler, a Split / Splitless injection port, and a Flame Ionization Detector (FID). The employed GC Column was an Agilent J & W 30 m x 0.319 mm. Part no. 1231334, Column ID No. 6170062, Liquid Phase DB-624, Film thickness: 1.8 µm, or equivalent.

The calibration standards were prepared in 2 mL glass vials sealed and crimped as follows: 20%, 40%, 60%, 80%, and 99.8% (or 1.55 mg/mL, 3.11 mg/mL, 4.67 mg/mL, 6.26 mg/mL, and 7.801 mg/mL OFP).

The Heater was set at 200 °C with a split ratio of 50:1. The initial column oven was set at 80°C and ramped at 1 °C/min, up to 89 °C. The total GC run time was 10 min. A flame ionization detector (FID) was used at 200 °C with air and hydrogen at 400 mL/min and 30 mL/min, respectively, plus a constant of Helium.

A calibration curve for the different OFP vials was prepared by plotting the listed concentration values for each calibration standard vs. the peak area data for the calibration standards. After having the calibration curve (R^2^=0.99 or more), the sample vials were run the same way. The concentration of OFP in the vials was then calculated by linear regression to determine the equation that describes the relationship between the calibration standard concentration listed by the manufacturer and the peak area produced by the chromatogram.

**Lipid content:** A specific, accurate reverse-phase HPLC method was developed to simultaneously determine DPPC, DPPE, and DPPE-MPEG-5K. An Agilent 1260 Infinity II LC system with an Agilent 1260 Infinity II ELSD detector (model G4260B) was used. An HPLC Column Acclaim Polar Advantage II (C18, 3 µm, 120 A, 4.6 x 150 mm) was employed with a non-isocratic mobile phase containing A: 100% Acetonitrile and B: 90% Methanol: 10% 60 mM Ammonium Acetate in different gradients. The flow rate was 0.8 mL/min. The elution order was: DPPE-MPEG5000, DPPC, DPPE, with a total run time of 20 min.

A calibration curve was calculated for the different phospholipids by plotting the listed concentration values for each calibration standard vs. the peak area data for the calibration standards. After having the calibration curve (R^2^=0.99 or more), the samples were run the same way. The concentration of each phospholipid was then calculated by linear regression to determine the equation that describes the relationship between the calibration standard concentration listed by the manufacturer and the peak area produced by the chromatogram.

**pH measurement:** The pH meter was calibrated with three standard pH buffer solutions of pH 4, 7, and 10. Three vials of each formulation were allowed to warm to room temperature and emptied into a clean glass container. A clean stir bar was added to the beaker, and the pH meter electrode was introduced until a stable reading was obtained. The pH of all formulations was within specifications of 6.2 to 6.8.

**Zeta potential:** The post-activation zeta potential of the MBs and PSMBs solutions was measured using the Malvern Zetasizer Nano ZS. Samples were diluted (Concentration factor = 1x10^-3^) in a 0.9% filtered NaCl solution and then assayed for zeta potential in a Folded Capillary cell (Malvern DTS 1070).

### Microthrombi Preparation

Fresh porcine whole blood in acid citrate dextrose (Lampire Biological Laboratories, Pipersville, PA) was mixed with 0.25 M CaCl_2_ (10:1) in type 1 borosilicate glass vials (Supelco Analytical, Bellefonte, PA) and incubated at room temperature for 3 h. The glass vials containing clotted blood were shaken on a dental amalgamator (Vialmix, Lantheus Medical Imaging) for 1-2 s to fragment the clot. To achieve a microthrombi of less than 200 µm, the suspension is passed through a 200 µm pore syringe filter. Finally, the size distribution was measured on the Coulter counter (Beckman Coulter Multisizer 4e, USA, 400 μm aperture).

### *In-vitro* Flow Loop Microthrombi Binding Assay

To study the binding affinity of MBs, FTMBs, PSMBs, and FTPSMB with the freshly prepared microthrombi, DiO dye-loaded formulations were used. We used our previously described *in-vitro* flow-loop system 20. Briefly, 1 mL of freshly prepared microthrombi of less than 200 μm diameter was introduced on a 40 μm Nylon mesh (fisher brand, USA) of the flow loop system. DiO dye-labeled formulations mentioned above at a concentration of 2x10^6^/mL were continuously infused by a syringe pump (Harvard Apparatus, USA) at a 1.5 mL/min flow rate for 10 min. In the control group, we used normal saline for perfusion, and the other parameters remained the same. Subsequently, the entire mesh was imaged at 4x magnification under a fluorescent microscope (OLYMPUS IX81) with an λex 644 nm; and λem 665 nm and analyzed using ImageJ software (National Institutes of Health).

### Rodent Hindlimb Model of MVO

The Institutional Animal Care and Use Committee of the University of Pittsburgh approved all experimental protocols. Young Wistar male rats (Envigo Labs, USA) weighing 275 ± 25 g were anesthetized with 3% isoflurane and maintained with 2%. The right external jugular vein was cannulated with a polyethylene 10 tubing intravenous catheter for infusion of DEFINITY^®^ for perfusion imaging. Polyethylene tubing was advanced from the right femoral artery into the abdominal aorta to administer microthrombi into the left hindlimb and subsequent therapeutic MBs or PSMBs infusion. The left hindlimb was shaved to allow for the positioning of the imaging and therapy transducers. A small animal's vital signs continuously monitor the heart rate, respiratory rate, and oxygen saturation (MouseOx, Starr Life Science, USA).

### Ultrasound Imaging

A Sequoia 512 clinical US imaging system (Siemens, Mountain View, CA, USA) was used to measure hindlimb muscle perfusion using a contrast-specific mode (CPS 7 MHz, 15L8 probe, Siemens). With the rat in the right lateral decubitus position, the imaging transducer was positioned lateral to the muscle. Burst-replenishment (MI=1.9 for burst, 0.2 for imaging) contrast-enhanced US (CEUS) perfusion imaging was performed during continuous intravenous infusion of DEFINITY^®^ via the internal jugular vein at 2 mL/h 34-36 at defined points of the therapy protocol. The video compression curve, dynamic range, and system gain were kept constant throughout the study.

### Contrast-Enhanced Ultrasound Perfusion Quantification

A region of interest (ROI) was selected on the CEUS images excluding the feeding vessels (microcirculation only), and the average video intensity in the ROI was quantified following the burst replenishment, up until the video intensity plateaued (typically < 30 s). It was previously reported by Wei et al. that the blood volume (A) and perfusion rate (AxB) could be estimated by fitting a mono-exponential function to the kinetics of video intensity (VI) using the equation 36: VI (t) = A(1-e-^Bt^). In the above equation, A is the maximal peak plateau video intensity, and AxB is the slope of the video intensity at t=0 and is consistent with the perfusion rate. A typical perfusion data and a fitted model have been previously described 22,34. CEUS analysis was performed offline on the cine loops obtained in CPS mode using custom MATLAB (version R2021a, MathWorks, USA) software.

### Sonoreperfusion Therapy

With the rat in the right lateral decubitus position, the imaging transducer was positioned anterior to the muscle, and imaging proceeded in the longitudinal plane along the muscle's midsection. Burst-replenishment imaging was performed as previously described 33, 34,35. Therapeutic US was delivered with a flat single-element immersion transducer 12.7 mm in diameter (A303S, Olympus NDT, USA), driven by an arbitrary function generator (AFG3252, Tektronix, USA) connected to a radiofrequency power amplifier (Model 250A250AM8, Amplifier Research, USA). The therapy transducer was oriented directly over the lateral aspect of the biceps femoris, orthogonal to the imaging transducer, such that the muscle was in the near field of the transducer and within the treatment area, as confirmed by visualizing MBs/PSMBs destruction in the perfusion image immediately after delivery of a therapeutic pulse. The therapeutic US was delivered at 1 MHz, 1.5 MPa peak negative pressure, 5000 cycles, and 3-5 s pulse interval (duty cycle 0.167% or less) for each of two 10 min sessions. The US field was calibrated with a 200 μm capsule hydrophone (HGL-0200, Onda, USA).

### Histopathology

Following ultrasound treatment, muscle tissues were collected from the hindlimb under the therapeutic ultrasound probe, stored in 10% Neutral buffered formalin (Fisher Chemical, USA), and sent to the HistoWiz, USA for processing and hematoxylin and eosin (H&E) staining.

### Statistics

Data were expressed as mean ± standard deviation. Statistical testing was performed using one-way ANOVA to compare the effects within the group and two-way ANOVA to compare the effects of the treatment group on two treatment times. Using Sidak's *post hoc* testing, multiple comparisons were analysed to report differences between groups. Analysis was performed using Prism (Version 9.3.1, GraphPad Software, LLC, USA). A *p*-value < 0.05 was considered statistically significant.

## Results

### Fibrin Binding Peptide Showed Increased Affinity Towards Fibrin Clots

**Figure 4:**
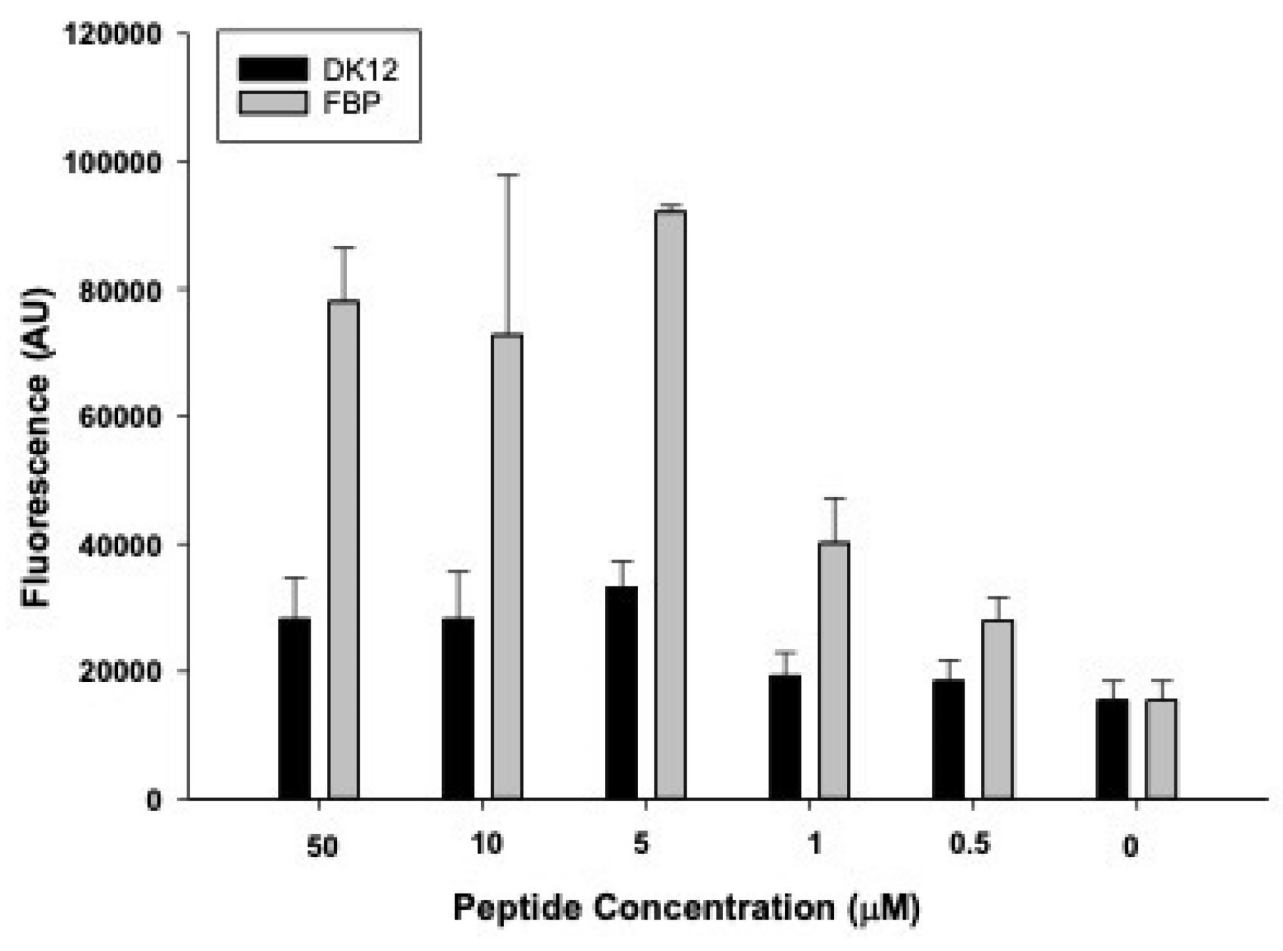
*In-vitro* fibrin binding affinity assay for fibrin thrombi with fluorescently labeled control peptide (DK12, black bars) and fluorescently labeled fibrin-binding peptide (grey bars).

The successful tagging of fluorescent dye (rhodamine) on FBP and control (DK12) peptides was confirmed by mass spectrometry. It was observed that when fibrin clots were incubated with 0, 0.5, 1, 5, 10, and 50 µM of FBP and DK12, a concentration of 5 µM of FBP showed significantly higher binding affinity and is in good agreement with the literature 32,33, which shows a kd = 1.7 µM. However, the DK12 peptide did not show binding to the fibrin clot at any given concentration in Figure 4.

### *In-vitro* Physical and Chemical Characterization

All the MBs and PSMBs parameters are summarized in Table 1. The results from the particle sizing analysis showed that the diameter of the MBs ranged from 820 to 880 nm, and the diameter of the PSMBs was ~150 to 250 nm. All formulations have more than the concentration specification for perfluorocarbon gas in the vial headspace before activation, which is ≥ 6.52 mg/mL. The concentration of each lipid and the total amount of the main lipids that form the shell are within the acceptance concentration range mentioned above. The pH of all formulations was within specifications (6.2 to 6.8). Finally, as expected, all formulations had a neutral net surface charge as evaluated by the zeta potential.

### FTPSMBs Have a Higher Binding Affinity to Porcine Microthrombi

**Figure 5:**
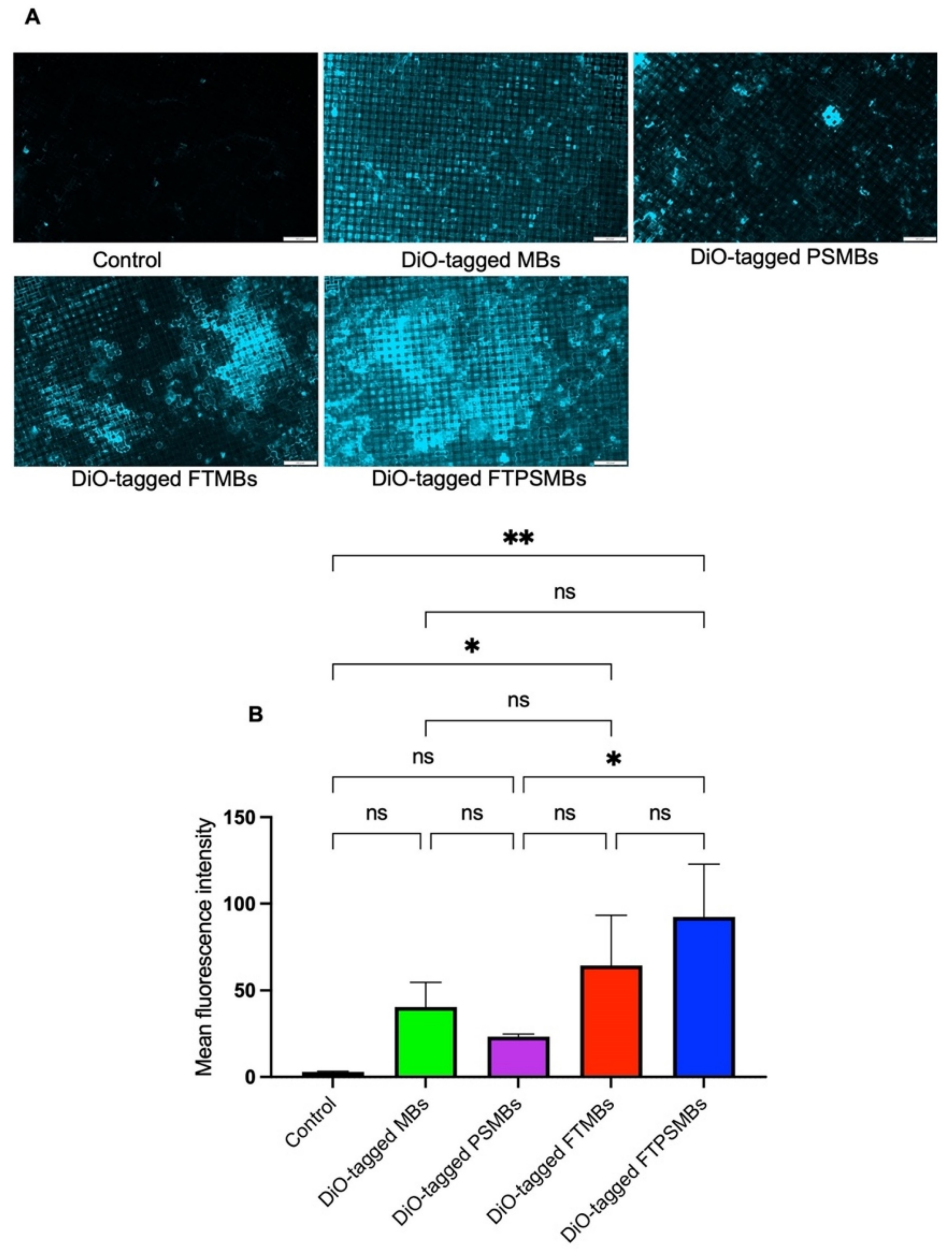
** In-vitro flow loop fibrin binding assay:** (A) Fluorescent images of the in-vitro flow loop mesh following 10 min of perfusion with saline (control), DiO tagged microbubbles (MBs), DiO tagged phase shift microbubbles (PSMBs), DiO tagged fibrin-targeted microbubbles (FTMBs), and DiO tagged fibrin-targeted phase shift microbubbles (FTPSMBs). (B) Bar graph representing the fluorescence intensity of three independent studies. Data expressed as mean ± standard deviation (*n*=3). *p<0.05, **p<0.001.

Following a confirmatory test of FBP affinity with the fibrin clot, we studied the binding affinity of DiO-tagged MBs, PSMBs, FTMBs, and FTPSMBs formulations with the freshly prepared porcine microthrombi using our *in-vitro* flow loop system (Figure 5). We observed the greatest fluorescent signal intensity on the mesh with FTSMBs compared to all other groups; this indicates a significantly higher thrombus binding affinity of FTPSMBs compared to the FTMBs, MBs, and PSMBs.

### FTPSMBs Outperformed FTMBs during Sonoreperfusion

**Figure 6:**
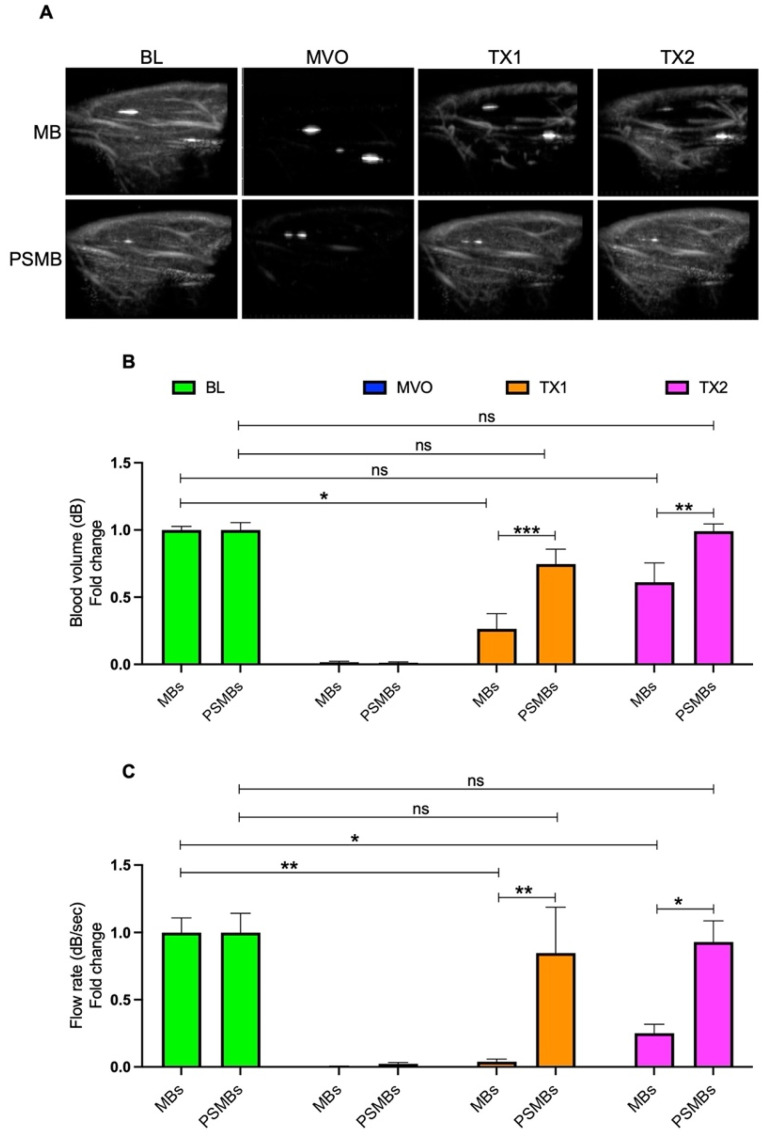
** Comparison between microbubbles (MBs) Vs. phase shift microbubbles (PSMBs).** (A) Contrast-enhanced ultrasound images of rat hindlimb. (B) Peak plateau video intensity which reflects the vascular cross-sectional area and is directly proportional to blood volume (dB). (C) Flow rate (dB/sec). Data expressed as mean ± standard deviation (*n*=5 for MBs and *n*=6 for PSMBs). *p < 0.05, **p<0.001, ***p<0.0001.

We then compared the therapeutic effects of each formulation in our rat hindlimb model of MVO. Initially, we compared untargeted MBs vs. untargeted PSMBs (Figure 6). We observed a significant increase in the blood volume (TX1; 0.26 ± 0.25 vs. 0.74 ± 0.27, *p <* 0.0005, TX2; 0.61 ± 0.32 vs. 0.99 ± 0.13, *p <* 0.007) and flow rate (TX1; 0.03 ± 0.04 vs. 0.84 ± 0.83, *p <* 0.004, TX2; 0.25 ± 0.14 vs. 0.92 ± 0.38, *p <* 0.02) following TX1 and TX2 with PSMBs compared to untargeted MBs.

**Figure 7:**
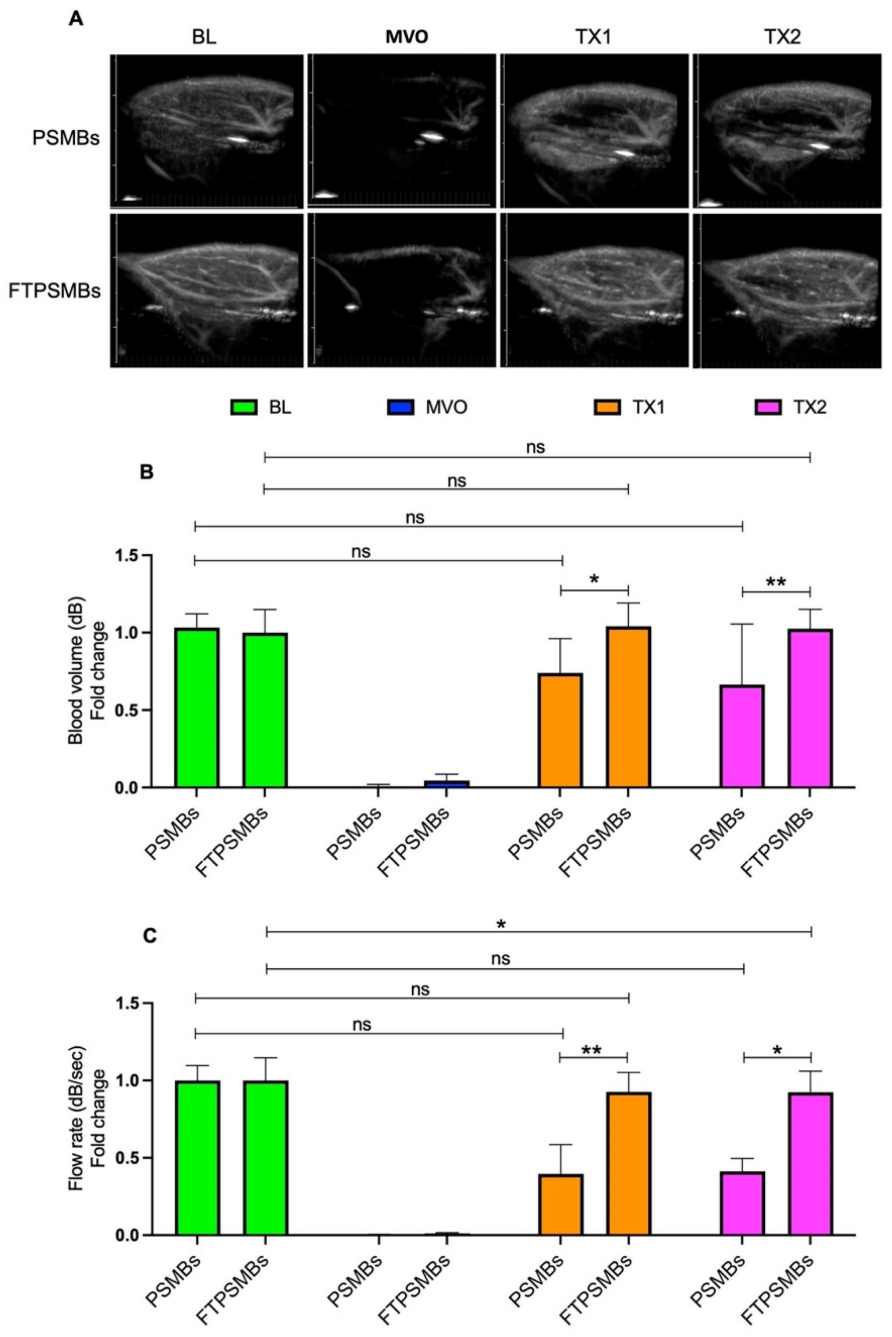
** Comparison between phase shift microbubbles (PSMBs) Vs. fibrin-targeted phase shift microbubbles (FTPSMBs).** (A) Contrast-enhanced ultrasound images of rat hindlimb. (B) Peak plateau video intensity which reflects the vascular cross-sectional area and is directly proportional to blood volume (dB). (C) Flow rate (dB/sec). Data expressed as mean ± standard deviation (*n*=6). Data expressed as mean ± standard deviation (*n*=6). *p < 0.05, **p<0.001.

We then compared untargeted PSMBs vs. FTPSMBs (Figure 7), and we observed a significant increase in the blood volume (TX1; 0.73 ± 0.22 vs. 1.04 ± 0.15, *p <* 0.02, TX2; 0.66 ± 0.39 vs. 1.02 ± 0.12, *p <* 0.006) and flow rate (TX1; 0.39 ± 0.46 vs. 0.92 ± 0.30, *p <* 0.009, TX2; 0.51 ± 0.53 vs. 0.50 ± 0.20, *p <* 0.01) following TX1 and TX2 with FTPSMBs.

**Figure 8:**
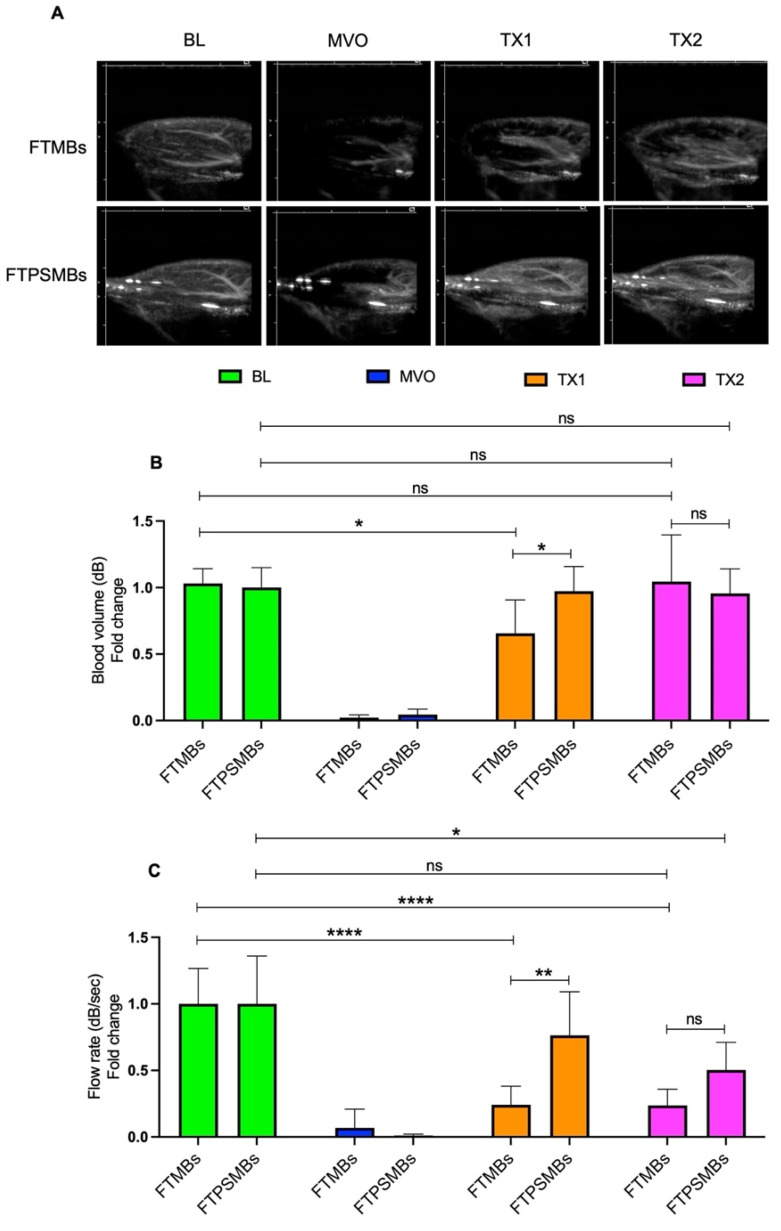
** Comparison between fibrin-targeted microbubbles (FTMB) Vs. fibrin-targeted phase shift microbubbles (FTPSMBs).** (A) Contrast-enhanced ultrasound images of rat hindlimb. (B) Peak plateau video intensity which reflects the vascular cross-sectional area and is directly proportional to blood volume (dB). (C) Flow rate (dB/sec). Data expressed as mean ± standard deviation (*n*=6). Data expressed as mean ± standard deviation (*n*=6). *p<0.05, **p<0.001 and ****p<0.0001.

Finally, we compared FTMBs vs. FTPSMBs (Figure 8). We found a significant increase in blood volume (TX1; 0.65 ± 0.25 vs. 0.97 ± 0.18, *p <* 0.02, TX2; 1.04 ± 0.35 vs. 0.95 ± 0.18, *p <* 0.88) and flow rate following TX1 (0.24 ± 0.14 vs. 0.76 ± 0.32, *p <* 0.00).

### Sonoreperfusion Therapy Cleared the Obstructed Microvasculature

**Figure 9:**
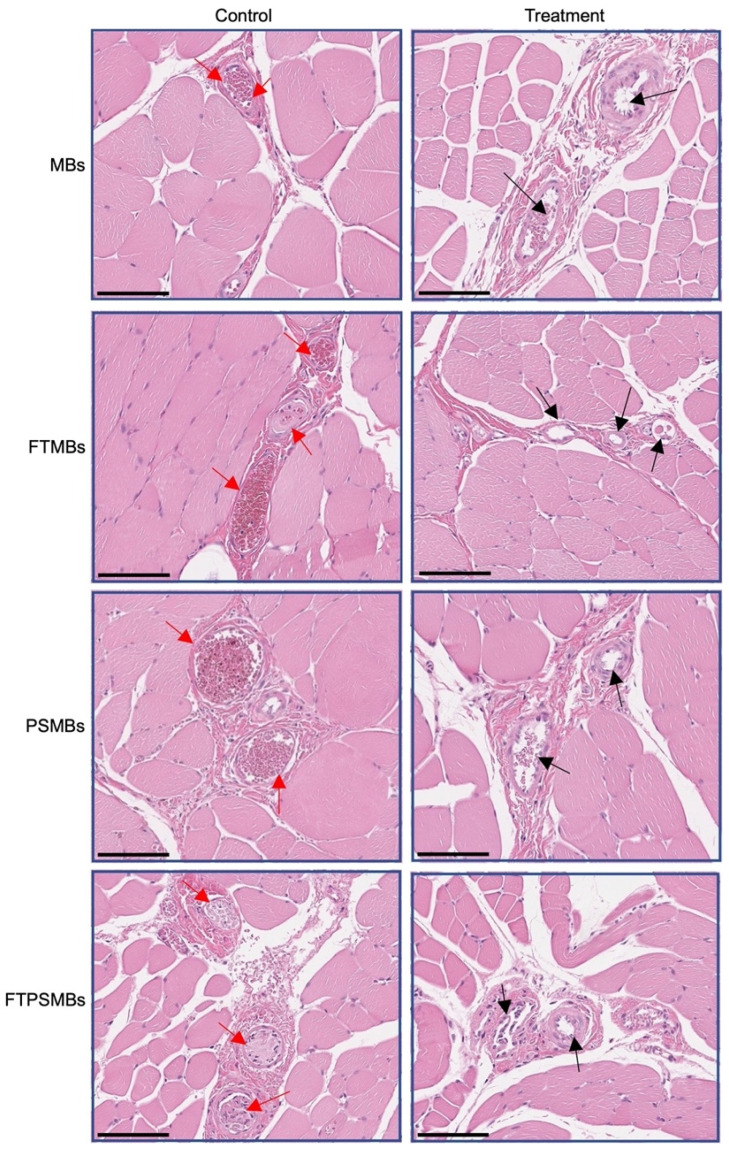
** Histology.** Hematoxylin and eosin staining of the rat hind limb muscle following ultrasound-targeted microbubble cavitation (UTMC) treatment with microbubbles (MBs), fibrin-targeted microbubbles (FTMBs), phase shift microbubbles (PSMBs), and fibrin-targeted phase shift microbubbles (FTPSMBs). Black arrows indicate microvascular patency compared to matching control; red arrows represent occluded microvessels. Scale bar = 200 μm.

Hematoxylin and Eosin images from the UTMC treatment region of the hindlimb showed a patent microvasculature in all the study groups (MBs, PSMBs, FTMBs, and FTPSMBs), in contrast to the persistently obstructed untreated control tissues (Figure 9).

## Discussion

MVO is highly prevalent in patients following PCI/stenting and causes significant morbidity and mortality 37. Therapeutic US for reperfusion is a growing and rapidly evolving subject of investigation. Several *in-vivo* studies suggest UTMC treatment strategies can restore flow in the occluded large epicardial coronary arteries 38-40. For example, Mathias et al. showed that US cavitation of intravenously administered MBs could increase early epicardial patency, reduce infarct size, and improve systolic function in patients with STEMI undergoing primary PCI clinical trial (NCT02410330) in a high-volume PCI center, where delays in mechanical reperfusion with PCI are often encountered due to limited catheterization lab availability 41.

However, MVO requires its own dedicated therapy. To that end, we previously demonstrated the efficacy of UTMC for treating MVO and recovering tissue perfusion 34. To further enhance microthrombi disruption and improve the efficacy of sonoreperfusion, our team developed novel FTMBs and FTPSMBs. For the first time, we report the robust therapeutic efficacy of FTMBs/FTPSMBs in an animal model for reperfusion of the obstructed microcirculation, an area routinely overlooked in interventional cardiology during epicardial recanalization.

Targeting fibrin within the microthrombi responsible for MVO allows greater proximity of the PSMBs/MBs to the microthrombi, a greater transmission of shear stress induced by PSMBs/MBs oscillation to the microthrombi 42, and as a result, more significant mechanical lysis of microthrombi 30. As expected, our* in-vitro* binding affinity assay results suggest an increase in the concentration of FBP (EP-2104R) increased the binding affinity for fibrin (Fig. 4). It is reported that EP-2104R binds equally to two sites on human fibrin (kd = 1.7 ± 0.5 µM) and has a similar affinity to mouse, rat, rabbit, pig, and dog fibrin. EP-2104R has excellent specificity for fibrin over fibrinogen (>100-fold) and for fibrin over serum albumin (>1000-fold). The peptide has been shown to bind in conditions of high shear stress in the heart 23,33.

We measured the size distribution of FTMBs/FTPSMBs using TEM. PSMBs are about 1/10 the size of standard MBs (average diameter 100-200 nm vs. 1-2 µm) and, owing to this smaller size, should penetrate deeper and in greater number into the microthrombi structure with nanometer-scale fibrin spacing (of note, PSMBs may also gain access to tissues beyond the vasculature and could deliver therapeutic compounds intracellularly) 30,31. TEM imaging of the FTPSMBs/PSMBs/MBs binding with fibrin clots revealed that 1) FTPSMBs permeate clot to a greater extent than non-targeted PSMBs and 2) non-targeted PSMBs penetrate deeper into clot than MBs. Our *in-vitro* fluorescent imaging results also demonstrated that greater thrombus adherence was achieved with FTPSBs versus all other groups. Together, these properties provide a more efficient lysis of the microthrombi during UTMC. Thus, our findings support our hypothesis; that is, owing to their smaller size and fibrin targeting, fibrin-targeted phase shift MBs UTMC yielded the most effective reperfusion of MVO.

Previously, selective fibrin targeting within the microthrombi was limited due to 98% structural similarities between fibrin and fibrinogen and a high concentration (2.5-3 mg/mL) of fibrinogen in the blood 43. However, in recent times specificity for fibrin over fibrinogen has been achieved by several techniques such as selective antibodies, small peptides, and tissue plasminogen activator (tPA) 44. Since fibrin is predominant in all vascular thrombotic obstructions, including arterial, venous, acute, and chronic, by microembolization, targeting fibrin can more effectively resolve MVO. The Fibrin peptide bioconjugate (DSPE-PEG5000-FBP) used in this study also showed more than 100-fold affinity towards fibrin over fibrinogen. Although several *in-vitro* fibrin binding affinity studies with fibrin-targeted microbubbles/nanodroplets, none were evaluated for thrombolysis in *in-vivo* models. This is the first study of its kind wherein we have successfully demonstrated the efficacies of FTMBs/FTPSMBs in an MVO model.

COVID-19 is associated with a significant risk of thrombotic complications ranging from microvascular thrombosis to venous thromboembolic disease and stroke 45. Microthrombi has been described as a fundamental cause of cardiac injury in COVID-19 patients 46. In one series of 40 hearts from patients dying of Covid-19, myocardial necrosis was found in 35%. Cardiac microthrombi were found in 64.3% of these hearts. We speculate that fibrin-targeted PSMBs might be used with UTMC in COVID-19 patients with microthrombosis and other thrombotic conditions to restore blood flow. UTMC with fibrin-targeted PSMBs merits further study and consideration for formal development as drug candidates to treat microvascular thrombosis and other thrombotic conditions.

### Limitations

We focus on the mechanical mechanisms of SRP on lysis of the microthrombi by MBs/PSMBs oscillations. The underlying biological mechanisms ideally should be elucidated, as well. For example, previous studies from our lab have demonstrated the release of nitric oxide from endothelial cells following in-house prepared standard caliber MBs and therapeutic US treatment to rescue MVO in the rat hindlimb 22. We have not correlated nitric oxide measurements with the observations reported herein. Other shear-mediated vasodilator pathways, such as endothelial-derived hyperpolarizing factor (EDHF), could also be considered. We did not quantify the efficiency of creating phase shift microbubbles from microbubbles with the cryo-condensation method, as the instrument we used only provided size information, not concentration information. Therefore, the dose of phase shift microbubbles was estimated from the microbubble concentration.

## Conclusions

In this study, we compared the efficacies of MBs, FTMBs, PSMBs, and FTPSMBs in a rat hindlimb model of microvascular obstruction. Our results demonstrate that using FTPSMBs causes the most rapid and complete reperfusion of MVO compared to all other agents tested. This recent body of work has opened a new paradigm on using FTPSMBs and ultrasound, shifting the focus from MBs to FTPSMBs for dissolving large thrombi to restoring microvascular perfusion following PCI. We demonstrate that FTPSMBs have a nanometer size range and fibrin affinity, enabling them to penetrate deeper into microthrombi, likely exerting more effective transmission of shear stress induced by oscillation, resulting in more significant mechanical lysis of microthrombi and ultimately enhancing reperfusion of MVO.

## Figures and Tables

**Figure 1:**
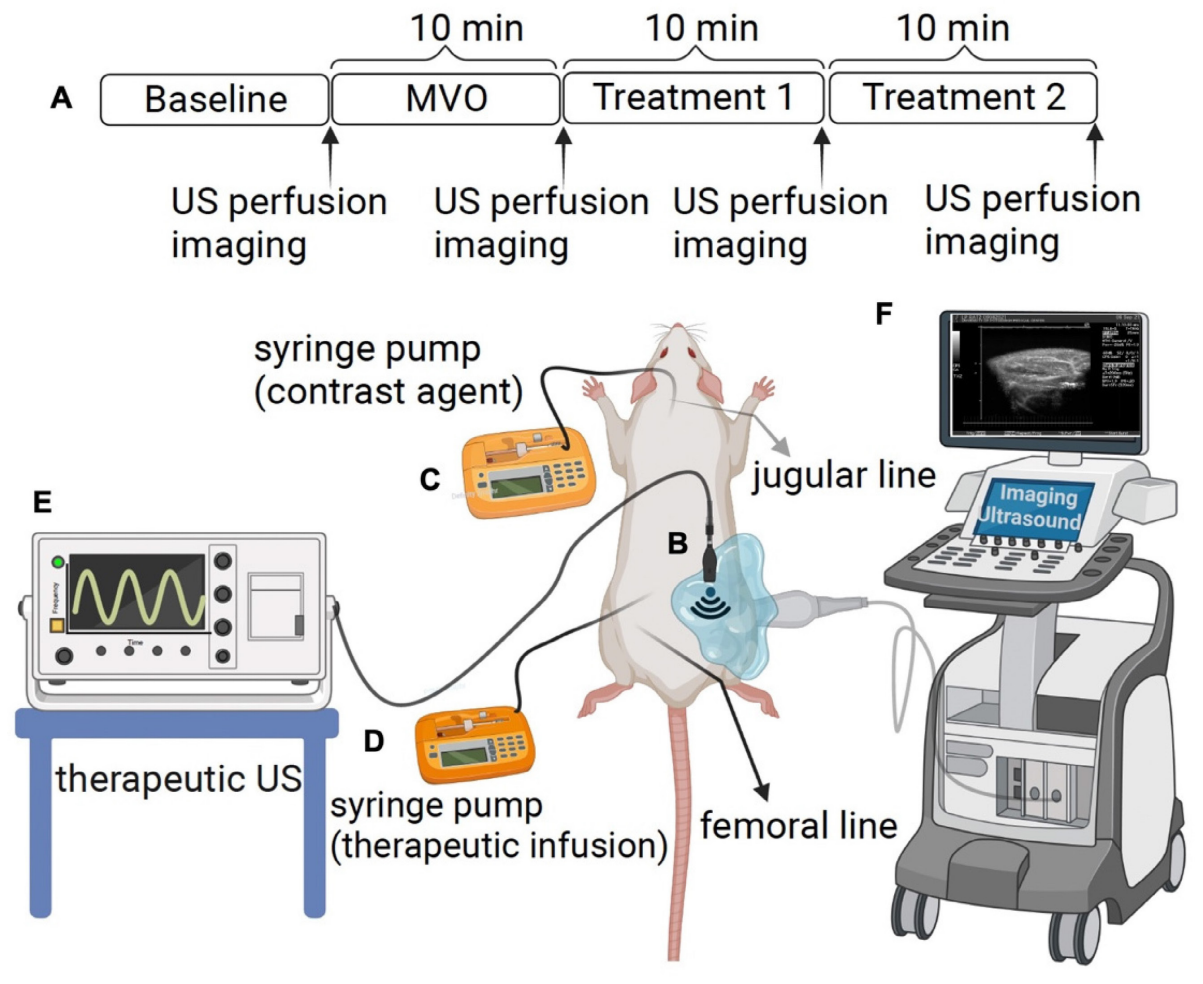
** Experimental setup.** (A) The rat hind limb microvascular obstruction (MVO) protocol includes an ultrasound (US) therapy and perfusion imaging time points. (B) A therapeutic probe was positioned perpendicular to the imaging plane of a clinical probe operated in contrast mode, which helps to guide and monitor US therapy. (C) Contrast imaging microbubbles (Definity^Ⓡ^) were infused via jugular access and were utilized for burst replenishment imaging to record perfusion. (D) A contralateral femoral arterial line was placed for microthrombi injection to create MVO and to deliver the therapeutic contrast agents for direct first-pass arterial delivery. (E) Therapeutic US. (F) Imaging US. (Figure created by BioRender).

**Table 1:** *In-vitro* characterization of non-targeted and fibrin-targeted formulations

Formulation		Particle Size (nm)	Concentration of OFP (mg/mL) (%)	Lipid Content (mg/mL)	Zeta Potential (mV)	pH
Non-targeted	MBs PSMBs	880.00 ± 11.11 245.51 ± 42.05	7.24 ± 0.21 88.28 ± 2.57	DPPE-MPEG-5K= 0.256 DPPC= 0.495 DPPE=0.044 Total lipid content= 0.795	-0.234	6.49
Fibrin- targeted	MBs PSMBs	820.00 ± 34.00 149.91 ± 33.15	6.73 ± 0.30 86.34 ± 3.91	DPPE-MPEG-5K= 0.295 DPPC= 0.417 DPPE=0.050 Total lipid content= 0.760	-0.611	6.45

## Abbreviations

AMI: acute myocardial infarction
MVO: microvascular obstruction
UTMC: ultrasound targeted microbubble cavitation
MBs: microbubbles
FTMBs: fibrin targeted microbubble
FTPSMB: fibrin targeted phase shift microbubble
US: ultrasound
STEMI: ST-elevated myocardial infarction
PCI: percutaneous coronary intervention
CEUS: contrast-enhanced ultrasound
ND: nanodroplets
SRP: sonoreperfusion
PSMB: phase shift microbubbles
tPA: tissue plasminogen activator
TEM: transmission electron microscope
TX1: treatment 1
TX2: treatment 2
OFP: octafluoropropane
FID: flame ionization detector (FID)
LV: left ventricle
HPLC: high-pressure liquid chromatography
MS: mass spectrometry
